# Optical Fiber Distributed Sensing Network for Thermal Mapping in Radiofrequency Ablation Neighboring a Blood Vessel

**DOI:** 10.3390/bios12121150

**Published:** 2022-12-08

**Authors:** Akbota Sametova, Sabit Kurmashev, Zhannat Ashikbayeva, Wilfried Blanc, Daniele Tosi

**Affiliations:** 1 School of Engineering and Digital Sciences, Nazarbayev University, Astana 010000, Kazakhstan; 2 National Laboratory Astana, Laboratory of Biosensors and Bioinstruments, Astana 010000, Kazakhstan; 3 Institute de Physique de Nice, CNRS UMR7010, Université Côte d’Azur, Avenue Joseph Vallot, 06108 Nice, France

**Keywords:** radiofrequency ablation, optical fiber sensors, distributed sensors, cancer thermotherapies, mini-invasive therapy

## Abstract

Radiofrequency ablation (RFA) is a minimally invasive form of thermotherapy with great potential in cancer care, having the capability of selectively ablating tumoral masses with a surface area of several cm^2^. When performing RFA in the proximity of a blood vessel, the heating profile changes due to heat dissipation, perfusion, and impedance changes. In this work, we provide an experimental framework for the real-time evaluation of 2D thermal maps in RFA neighboring a blood vessel; the experimental setup is based on simultaneous scanning of multiple fibers in a distributed sensing network, achieving a spatial resolution of 2.5 × 4 mm^2^ in situ. We also demonstrate an increase of ablating potential when injecting an agarose gel in the tissue. Experimental results show that the heat-sink effect contributes to a reduction of the ablated region around 30–60% on average; however, the use of agarose significantly mitigates this effect, enlarging the ablated area by a significant amount, and ablating an even larger surface (+15%) in the absence of blood vessels.

## 1. Introduction

Radiofrequency ablation (RFA) is a form of medical thermotherapy that makes use of a miniature applicator, incorporating one or multiple electrodes, with the goal of delivering a difference of potential from a mid-frequency oscillator (50–450 kHz) to the tissue under treatment [1,2,3]. RFA has been established as a common procedure for the correction of cardiac arrhythmia [4] and interventional pain management [5], exploiting its capability of ablating small regions; after the 2000s, however, it has been increasingly used in cancer care for the removal of solid tumors such as hepatocellular carcinoma [1,6].

RFA has been established as a mini-invasive cancer treatment, an alternative to its counterparts operating at higher frequencies such as microwave [7] and laser ablation [8]. When using lasers as ablation sources, the light is delivered through a large-core fiber, but its capability for ablation is limited as the light is absorbed in a small teardrop-shaped region [9]; on the other hand, in microwave ablation operating at a few GHz, the applicator serves the purpose of a near-field antenna matching the impedance of the tissue, and therefore, it has a lower potential for miniaturization [10]. RFA provides a treatment that combines the miniaturization of the applicator and its tip electrodes with the localization of the bio-heating process in the surrounding tissue [11], as well as allowing the ablation of large volumes. Recent studies, in addition, have shown how the use of gold [12] and silver [13] nanoparticles might significantly expand the treated area. For example, Ashikbayeva et al. reported a 102% increase in the ablated surface area when using green-synthesized Ag nanoparticles in an agarose gel, and a 72% increase when using agarose only. Agarose gel is widely used in biomedicine material; it has biocompatible, antimicrobial characteristics, and demonstrates good heating performance in terms of temperature, heat distribution and the short time it takes to reach cytotoxic temperature ranges [13]. Overall, RFA allows a rapid (30–120 s) treatment, capable of reaching cytotoxic (>42 °C) and instantaneous cell mortality (>60 °C) temperature values [14] within a few seconds from the start.

One of the ongoing technological challenges is the estimation of the effects of large blood vessels in proximity to the treated area to RFA treatment, and how to compensate for such limitations [15,16]. According to De Vita et al. [17], the presence of a blood vessel affects the thermal treatment mainly due to the heat-sink effect, due to heat convection, which causes a reduction and an asymmetry of the heating pattern as the temperature near the vessel approaches ~60 °C. Additional effects include blood perfusion through the vessel and changes in tissue impedance [18].

Numerical studies have been employed to estimate the heat-sink effect. Huang [15] reported a simulative framework, modifying the bio-heat equations for blood vessels located 2–10 mm away from the electrode (parallel and perpendicular to the RF applicator) and estimating a reduction of the ablated volume of 5–23%. A similar study was carried out by Tungjitkusolmun et al. in 2002 [19] for a bifurcated vessel ablated by a multi-tip device, while a more recent investigation from Vaidya et al. [20] reported the dynamic effects of blood perfusion through the vessel and its cooling effect in time-varying heating equations.

However, the large variability of results obtained in RFA—due to the different bio-electrical properties of the tissues [21], RFA parameters including the typology of the applicator [22], and the possibility of using gels and nanomaterials to affect the electromagnetic performances [23]—demand a system that is capable of estimating the effects experimentally and in real time, maintaining the same mini-invasive form factor of RFA.

The studies from Puza et al. [24] and Pillai et al. [25] aimed at the experimental evaluation of heat-sink effects during thermal ablation, using electrical thermocouples (TCs) ex vivo in animal models. More recent investigations from De Vita et al. have reported the experimental analysis of the heat-sink effect in laser [18] and microwave [17] ablation using fiber Bragg gratings (FBGs). FBGs are less invasive compared to TCs, and they improve in terms of accuracy and response time; most significantly, the possibility of using inline arrays of FBGs with narrow spacing allows the rendering of the temperature along the optical fiber hosting the gratings, improving the spatial resolution [26].

Despite the different working principles, both TCs and FBGs require the accurate positioning of the sensing devices in the tissue in order to provide the exact measurement of the heat-sink effect [17]. In order to overcome this limitation, distributed sensing networks have been utilized [13,27]: in this case, rather than using an optical fiber as the sensing element, the temperature detection is encoded in the back reflection due to the Rayleigh scattering that occurs in all optical fibers, including single-mode telecom fibers [28].

The first work in applying distributed sensing, using the optical backscatter reflectometry (OBR) principle, was reported by Macchi et al. [29]. This system achieves the best thermal rendering when using the scattering-level multiplexing (SLMux) configuration: in this case, the sensing elements are replaced by high-scattering fibers, having the core doped with Mg-silicate nanoparticles [30]. Such fibers are designed to match the size and compound of single-mode fibers, but yield a scattering increment of >30 dB; the SLMux enables the simultaneous and distributed detection of each fiber with sub-centimeter resolution, and can provide 2D [27] and 3D [31] temperature sensing in thermotherapies.

In this work, we provide an ex vivo evaluation of the heating dynamics in RFA using a setup that mimics a blood vessel in the proximity (10 mm) of an RFA applicator. Thermal data have been acquired using a distributed sensing network with six fibers, forming a grid with 2.5 × 4 mm spatial resolution over 800 mm^2^ surface. Furthermore, we provide a possible solution to overcome the heat-sink limitation, through the injection of agarose gel in the tissue which facilitates impedance matching and improves the treated surface. The method hereby reported allows real-time operation, and is suitable for in vivo operation, owing to the biocompatibility and microscopic form factor of the optical fibers.

The proposed method provides a 2D surface thermal mapping that allows the real-time detection of thermal patterns in situ in the proximity of the thermal ablation applicator. The presence of neighboring blood vessels causes an alteration of the temperature distribution, depending on the size, position, and flow speed occurring in the vessels [32]. While previous works based on FBG sensors are able to render these thermal effects only if the FBG array is tactically placed in proximity of the blood vessel itself [17], the presence of a multi-fiber distributed sensing network enables sensing at a much larger scale, helping to identify the heat-sink effects but also to render the whole thermal distribution on the ablation plane. The results presented in this paper extend over 15 experiments, in order to verify the whole set of conditions for thermal ablation procedures based on RFA.

## 2. Experimental Setup

### 2.1. Radiofrequency Ablation Setup

**Figure 1:**
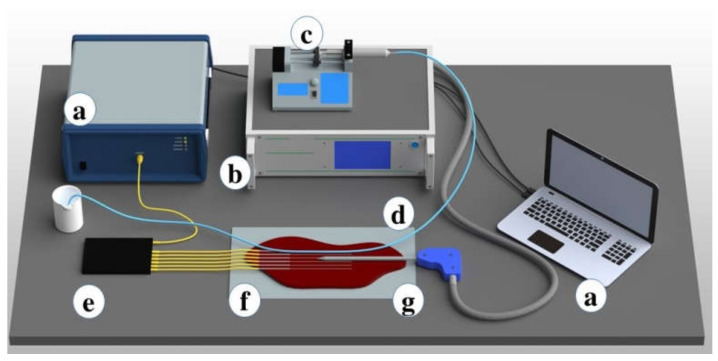
Schematic view of the setup for the radiofrequency ablation of the phantom. The setup includes (**a**) OBR and data logging; (**b**) customized RF generator for the ablation; (**c**) the syringe pump; (**d**) medical tube positioned in parallel to the active electrode; (**e**) six optical fiber sensing arrays; (**f**) parenchymal tissue; and (**g**) an applicator of the RF generator inserted in tissue between fibers 3 and 4.

The thermal ablation setup is illustrated in Figure 1. The setup consists of three main units: (1) the distributed temperature sensing system; (2) the RF applicator with delivery system; (3) the blood vessel system with its perfusion. The temperature sensing system is based on an optical backscatter reflectometer (OBR, Luna 4600, Roanoke, VA, USA) that was used for the distributed interrogation. The OBR was connected to the temperature sensing array consisting of six Mg-silicate nanoparticle-doped fibers, fabricated according to [33,34] and spliced to single-mode fibers. The system makes use of an optical splitter to multiplex between multiple sensing channels, each acquired simultaneously, according to the scattering-level multiplexing principle [35]. The second unit is the source of electromagnetic energy, a customized RF/MW Hybrid Generator (LEANFA S.r.l., Ruvo di Puglia, Italy). The RF waves were delivered to the liver phantom by an applicator shaped for percutaneous insertion that has an active electrode (AE) made of brass mounted on the tip of a device with a 160 mm length and 3 mm diameter. The RF power was set at 50 W and the generator frequency was 450 kHz. Moreover, the instrument controls the rise of tissue impedance by means of an embedded impedance meter and terminates the ablation process as the tissue resistance reaches 800 Ω (safe mode operation). These parameters were kept constant for all the experiments. Finally, the third part is a perfusion system with a medical tube (catheter) of 60 cm in length and an inner diameter of 4 mm connected to the syringe pump (Legato 100, KD Scientific, Holliston, MA, US) operating at a flow rate of 24 mL/min, which is similar to the blood flow rate. A 60 mL syringe filled with water was placed on the syringe pump to mimic the blood circulation near the ablation area.

**Figure 2:**
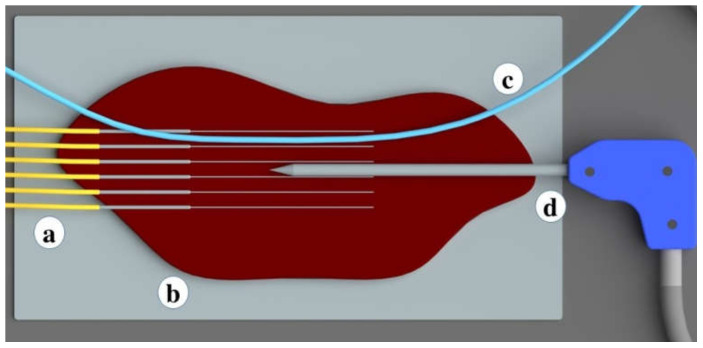
Zoomed schematic of the positions of the optical fibers, medical tube, and the active electrode. The schematic includes (**a**) six optical fibers positioned in y-axis in parallel at 4 mm distance; (**b**) a bovine liver; (**c**) a medical tube mimicking the blood vessel positioned in parallel to optical fibers and active electrode; and (**d**) RF applicator (active electrode).

**Figure 3:**
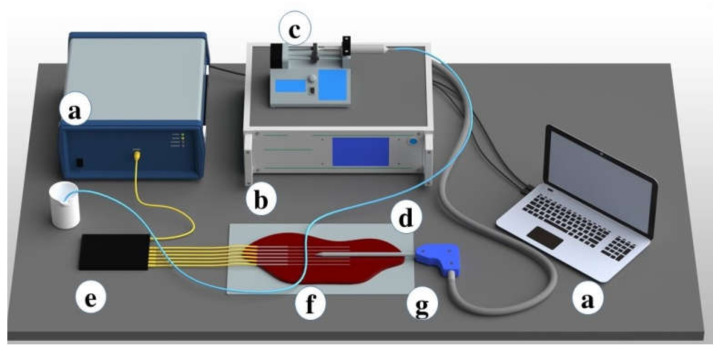
Schematic overview of the radiofrequency ablation setup. The setup includes (**a**) OBR and data logging; (**b**) RF generator; (**c**) the syringe pump; (**d**) a medical tube located perpendicularly to the optical fibers; (**e**) six optical fibers to register the temperature change; (**f**) parenchymal tissue; and (**g**) an applicator of the hybrid generator inserted between 3rd and 4th optical fibers.

**Figure 4:**
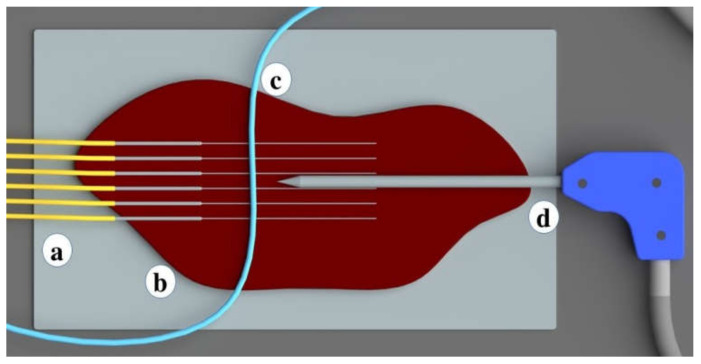
Zoomed representation of the position of medical tube mimicking the blood vessel and temperature sensing optical fibers on the tissue. The medical tube and optical fibers are located perpendicularly to each other on x-y plane. The setup consists of (**a**) optical fibers; (**b**) a bovine liver; (**c**) vessel located in parallel; and (**d**) an applicator.

Medical tubes convenient for the perfusion of the liquid were used to simulate the heat-sink effect during the ablation procedures. Medical tubes were positioned on the tissue surface in two ways: in parallel (Figure 1 and Figure 2) and in perpendicular (Figure 3 and Figure 4) with respect to the location of the active electrode. This setup based on tubes mimicking the blood flow originated from the previous reports by De Vita et al. for the investigation of heat-sink effects in microwave [17] and laser [18] ablation.

### 2.2. Distributed Sensing Network

The temperature sensing network was constructed using six nanoparticle-doped optical fibers spliced to standard single-mode fibers (SMF-28); different lengths of SMF spans have been used as delay lines to disambiguate each sensing fiber. The fibers used for temperature sensing are silica fibers drawn for preforms prepared by the MCVD (Modified Chemical Vapor Deposition) process [36]. Mg-silicate nanoparticles were obtained by adding erbium (III) chloride hexahydrate and magnesium chloride during the doping solution step [34]. The obtained optical fibers have core and cladding diameters of 10 µm and 125 µm, respectively, and the same temperature coefficient as SMF fibers (10.4 pm/K).

The sensing optical fibers were connected to the OBR using a coupler to collect the data from backscattered light. The OBR works on the principle of Rayleigh backscattering and records the scattered light from each point of the optical fiber. The collected data generates a pattern of backscattered light based on the location along the optical fiber.

The OBR operated at the following parameters: the wavelength range is 43.01 nm, the sensing range is 1.213 m, and the gauge length is 0.499 m.

### 2.3. Thermal Ablation Experiments

Commercially purchased bovine liver was used as a liver phantom. The liver tissue was stabilized at room temperature (22 °C) for 6–8 h before the experiments. The initial temperatures were measured by a contact thermometer (IKA ETS-D5, Staufen, Germany). Then, the liver phantom was positioned on the surface of the ground plate of the RF generator, called the passive electrode.

The temperature sensing system was fixed on the surface of the liver phantom to record the temperature changes during the RFA. The sensing system containing the multiplexed array of optical fibers was intended to cover the majority of the ablation area. The distance between fibers was 4 mm in the x-axis, while the distance between the tip of the applicator and the vein-mimicking tube was 1 cm for the parallel location setup, and 1.5 cm for the perpendicular location setup. The parallel and perpendicular location of the tube is depicted in Figure 2 and Figure 4, respectively. The heating through RF ablation lasted for 50 s, followed by 50 s of cooling. The applicator was positioned between the third and fourth fibers.

All the experiments were repeated three times with the pristine tissue and liver phantom injected with 100 µL of 0.2% agarose gel. Agarose gel was prepared by dissolving agarose powder in Tris-acetate-EDTA buffer.

Overall, fifteen experiments were carried out for parallel/perpendicular vessel location, pristine/agarose-introduced conditions, and without a vessel to compare the results. Water and agarose gel were also maintained for 3 h at room temperature and injected by syringe all around the applicator tip, six optical fibers, and vessel tube.

We remark that, in line with the requirements for thermal ablation, the form factor of the fiber sensors is compatible with disposable use; both the SMF fibers and the high-scattering fibers are produced and spooled over hundreds of meters in length, and SMF fibers are compliant with the ISO 10993 standard of biocompatibility.

### 2.4. Thermal Maps Evaluation

In this study, data processing went through the following steps. We first ran all the data obtained from Luna OBR4600 (Luna Inc., Roanoke, VA, USA) through a threshold algorithm to identify the precise location of each fiber on the data array. Then, we found the coordinates of the peak temperature in this region which represents the tip of the heating needle. Next, the arrays of 20 data points around the tip were extracted and interpolated. By repeating the same process on each data array, sampled every second, dynamic temperature maps were derived, and from these, the main metrics such as temperature peak, cytotoxic area (temperature > 42 °C), and ablated area (temperature > 60 °C) were derived.

## 3. Experimental Results

### 3.1. Thermal Maps

**Figure 5:**
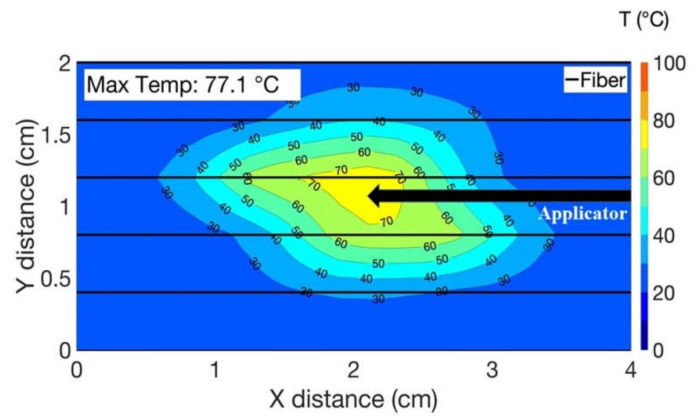
2D thermal map presenting the heating outcome of the phantom injected with agarose gel during RFA and with the blood-vessel-mimicking medical tube located in parallel to the optical fibers.

Figure 5 presents the results of the heating during RFA of the liver phantom mediated with agarose gel in the presence of the blood-flow-mimicking vessel. The vessel containing the water and running at 20 mL/min flow rate was positioned on the tissue surface in parallel to the AE. The data was acquired by the linear interpolation over 20 data points along the longitudinal axis from all six fibers and reported in the form of a thermal map. Thermal maps show the change in temperature over the area that has been ablated over time. The acquired thermal map indicated the temperature increased up to a maximum value of 70 °C in the vicinity of the AE and lowered up to 30 °C in the periphery area in the presence of the blood-flow vessel. The position of the applicator displayed in Figure 5 has been maintained for the whole set of experiments.

**Figure 6:**
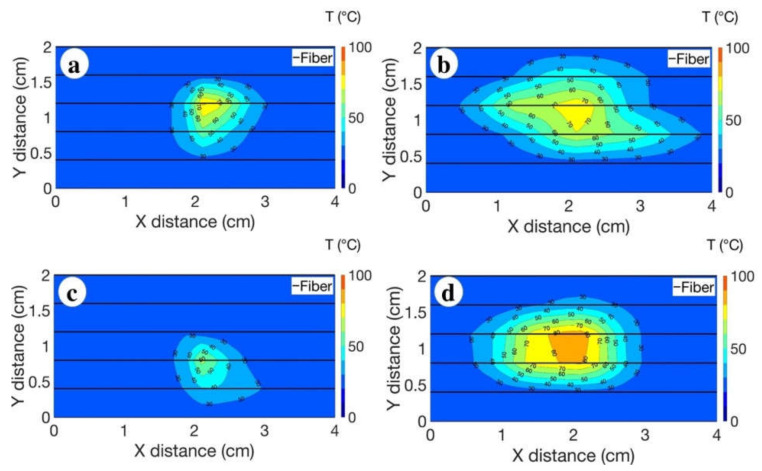
Thermal maps showing the evaluated heating effects in four cases: (**a**) pristine liver tissue with the vessel located in a parallel position; (**b**) the tissue introduced with the agarose gel, with the location of the vessel in parallel to the optical fibers; (**c**) pristine tissue with the vessel located in a perpendicular direction; (**d**) the tissue introduced with the agarose gel, with the vessel positioned in a perpendicular direction. The charts report the isothermal curves, sampled at each 10 °C temperature differential.

Four comparative thermal maps reported in Figure 6a–d represent the heat distribution pattern depending on the location of the blood-flow-mimicking vessel. The higher temperature values during RFA were detected during the ablation of the tissue with the vessel located in a perpendicular direction to the AE (Figure 6c,d), compared to the heating outcome achieved with the parallel-oriented blood flow (Figure 6c,d) system. Moreover, the administered agarose gel added to the heating performance and increased the temperature up to 80 °C (Figure 6d).

The collected data enabled the reconstruction of a video of the RFA in the tissue treated with agarose, with the blood-flow-mimicking system positioned in parallel and perpendicular to the AE. The full video of all the experiments demonstrating the heating patterns in the form of 3D thermal maps are shown as Supplementary Material that can be accessed online via the included link in Data Availability Statement.

**Figure 7:**
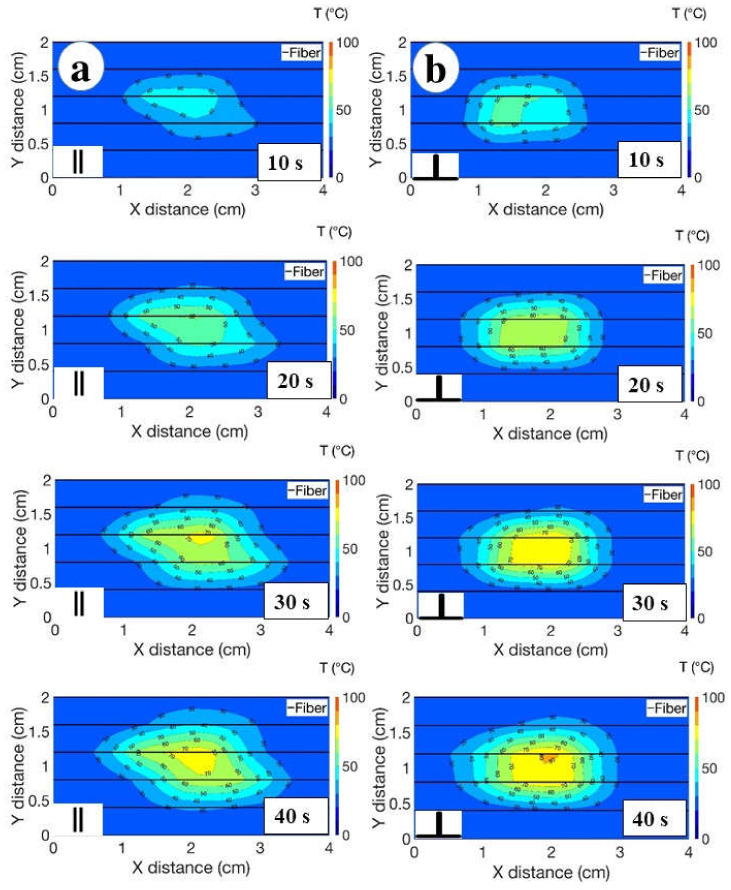
The cinematic view recorded during the RFA of the liver tissue injected with agarose over 40 s for two heating scenarios: (**a**) the parallel location of the blood vessel and optical fibers; (**b**) the perpendicular location of the blood vessel and optical fibers. The thermal map was registered every 10 s. Different colors on the 2D map stand for the different temperature values. The two dimensions illustrate the fiber location parallel to the x plane. The maximum temperature was recorded at 40 s of the ablation. The charts report the isothermal curves, sampled at each 10 °C temperature differential.

The ablation process was rapid and lasted for 50 s for both scenarios, the parallel and perpendicular position of the vessel. The cinematic view of the temperature change at every 10 s was recorded for both cases and reported in Figure 7. The obtained results demonstrated that the vessel located in parallel to the AE contributes more in the cooling of the temperature during RFA.

**Figure 8:**
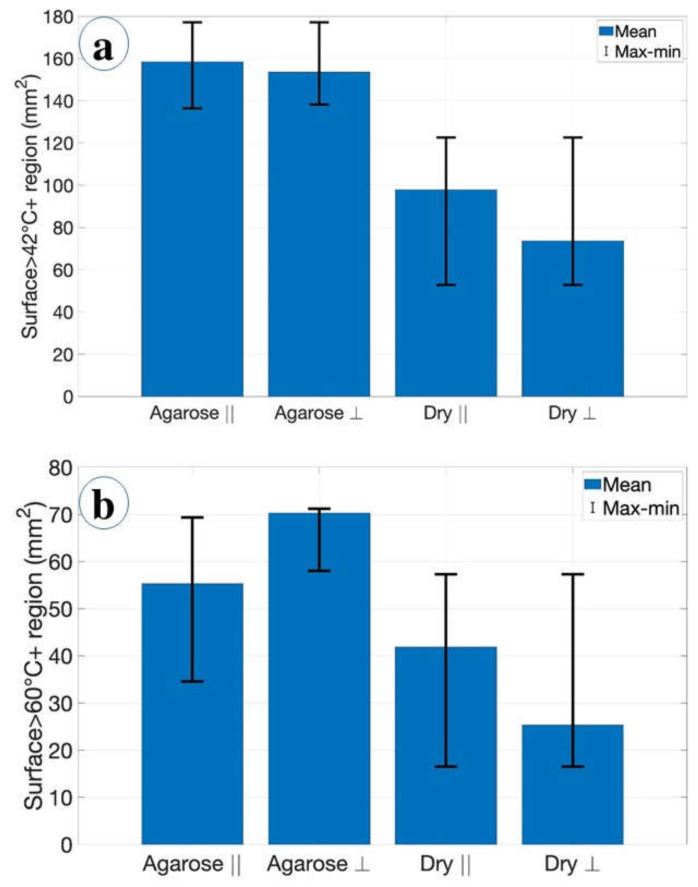
The cytotoxicity chart estimated for the area of ablation on tissue at several parameters: (**a**) the ablated area at 42 °C for the pristine tissue and tissue treated with agarose in parallel and perpendicular positions as labeled in the chart. Lines indicate an average of three experiments and error lines are equal to the standard deviation for 42 °C; (**b**) estimation of the ablated area for each condition of the pristine tissue and tissue with the injected agarose gel in parallel and perpendicular positions. Lines show the error bars obtained as a function of an average of three experiments and error lines are equal to the standard deviation for 60 °C.

The area of ablation during RFA at 42 °C (Figure 8a) and 60 °C (Figure 8b) was analyzed for pristine tissue and tissue treated with agarose in the parallel and perpendicular positions of the vessel. The relevance of the cytotoxic temperature range between 42 °C and 60 °C was analyzed in the work of Sapareto et al. [14], demonstrating that the coagulation of proteins occurs around these temperature values for rapid ablation phenomena.

Figure 8a shows the maximum area of ablation with the mean and maximum values. The area of ablation was estimated as 1.6 cm^2^ when the tissue was introduced with agarose gel, while the maximum area of ablation at 60 °C was 0.7 cm^2^ with the perpendicularly positioned vessel as demonstrated in Figure 8b.

**Figure 9:**
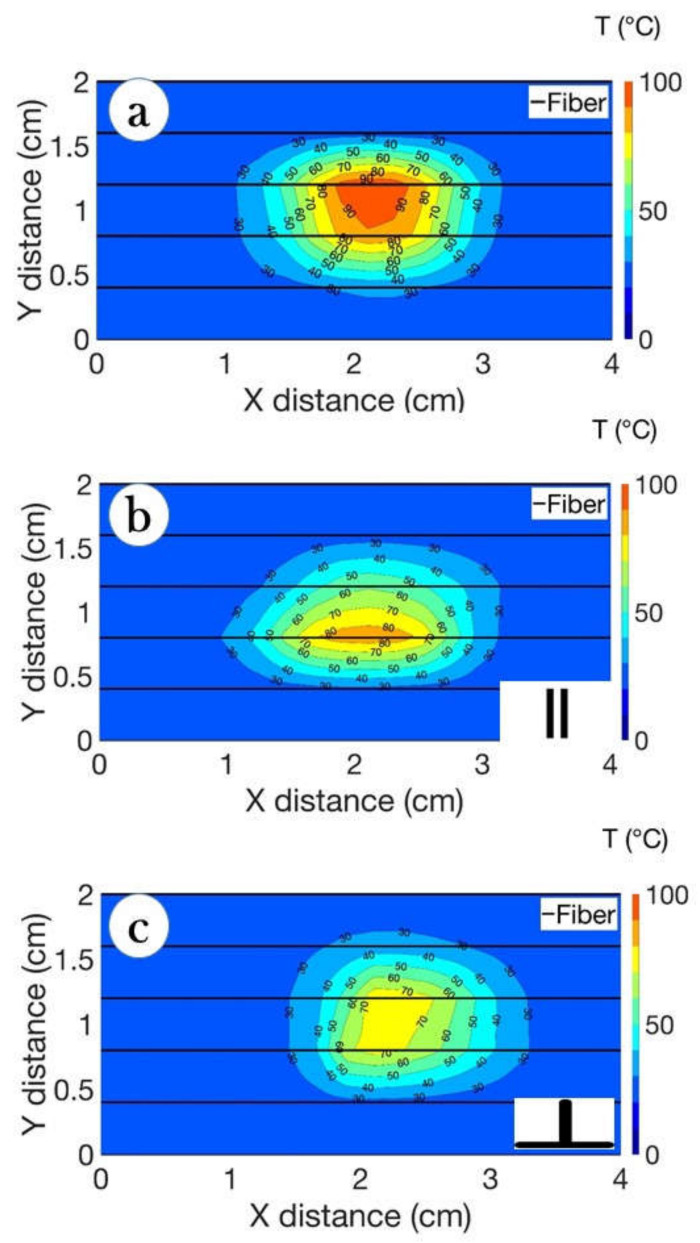
Thermal maps illustrate the temperature difference between cases: (**a**) pristine tissue without vessel placement; (**b**) tissue injected with agarose gel and vessel located in parallel to optical fibers; (**c**) tissue injected with agarose and vessel located in perpendicular to the optical fibers. The displayed maps report the temperature distribution observed for the ablation peak.

Figure 9 shows the thermal maps obtained in three conditions: pristine ablation (without the presence of any blood vessel), and with tissue injected with agarose and a blood vessel positioned in parallel and perpendicular, respectively, to the applicator. We can see the thermal effects acting on the temperature distribution, as the maximum temperature decreases while the heat distributes in a lesser penetrative way through the peripheral sides of the tissue.

### 3.2. Maximum Temperature

**Figure 10:**
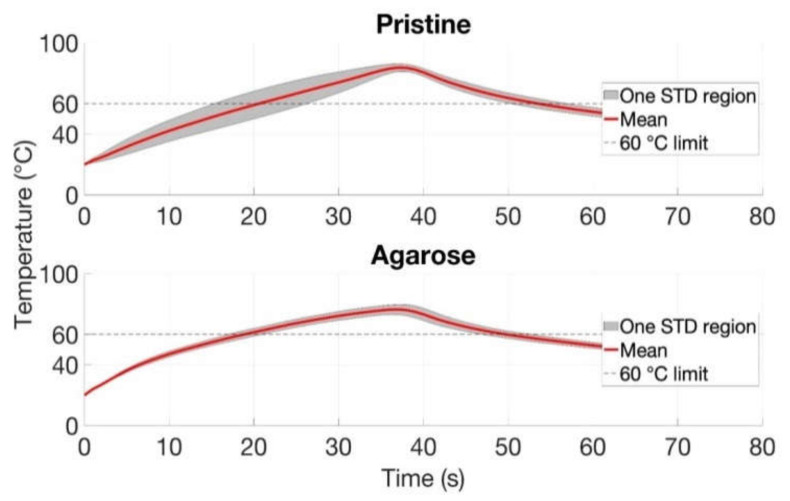
The maximum temperature values achieved during RFA over time in the presence of blood-mimicking system oriented in parallel to AE. The maximum values were reached at 40 s followed by a gradual decrease in both pristine and agarose-administered tissues. The analysis included the mean and standard deviation values evaluated over three trials for each case.

Figure 10 reports the maximum temperature with mean and standard deviation values reached during RFA for the pristine tissue and tissue with the injected agarose solution parallel to the vessel. Figure 10 presents the temperatureincrement trend with the vessel located in parallel on agarose-assisted tissue and pristine tissue for three repeated trials. The time to reach 60 °C was twice as short in the case of the ablation of the tissue injected with agarose gel compared to the pristine tissue, which took 10 s and 20 s, respectively. Figure 10 shows the standard deviation region that the grey-shaded area represents. Agarose-mediated experiments with parallel vessels showed a thicker trend in comparison with the pristine tissue, which has a greater extension.

Agarose-mediated experiments demonstrate the highest repeatability in the experiments with the perpendicularly located vessel; however, the time to reach 60 °C was almost similar (20 s) for both conditions.

**Figure 11:**
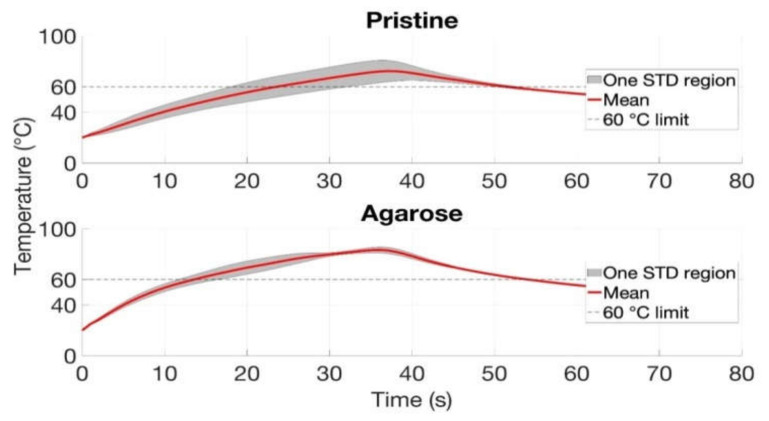
The maximum temperature values achieved during RFA over time in the presence of blood-mimicking system oriented perpendicularly to AE. The maximum values were reached before 40 s followed by a gradual decrease in both pristine and agarose-injected tissues. The analysis included the mean and standard deviation values evaluated over three trials for each case.

Figure 11 shows the trend in the temperature growth for the perpendicularly oriented vessel with agarose-mediated and pristine conditions. It can be clearly seen that the standard deviation region is wider in the case of the pristine tissue for three repetitions, while the agarose-mediated results show the same trend for all experiments. However, the mean time to reach 60 °C is similar for both cases, 20 s. A further visualization of the percentage change of the cytotoxic and ablated area has been listed in the Supplementary Data, available online in Data Availability Statement.

## 4. Discussion

Thermal ablation therapy is known as an efficient tumor treatment technique. However, the efficiency of the thermal therapy is significantly affected by the presence and the amount of blood flow to the tumor and neighboring area, whichcauses a cooling effect. This effect is called the “heat-sink effect”, which describes the relationship between the liver blood flow with its cooling features and the formation of a lesion. The accurate prediction of the ablation zone is hindered by the heat-sink effect affecting the heat distribution [32]. Hence, the precise estimation of the coagulation zone is vital for the implementation of RFA in clinical trials. An optical fiber temperature sensing system enables precise temperature monitoring with an accurate evaluation of the ablated area. It is also noteworthy that while the optical fibers used in sensing are very small and do not alter the temperature distributions, thermocouples used in several works (e.g., [37]) are themselves a cause of heat-sink dissipation as they heat faster than the tissue.

The increment of the temperature during RFA can be affected by several parameters such as the location of the blood vessel and its distance from the tumor [31]. The thermal maps presented in Figure 6 show that the vessel located in a parallel position at a distance of 1 cm has a higher impact on the temperature cooling compared to the perpendicularly located vessel at a distance of 1.5 cm, indicating that the distance of the vessel from the tumor is an important parameter that can affect the heating performance.

As demonstrated in Figure 7, Figure 8 and Figure 9, the bigger difference in ablation diameter in the case of parallel orientation of the blood flow compared to perpendicular means that there is more heat loss close to the vessel and AE, which can be attributed to the bigger area covered by the vessel located in parallel with the AE.

As shown in Figure 9, the “heat-sink effect” limits the increment of the ablation area during RFA. The estimated surface area in the x-plane during the ablation of the pristine tissue and the tissue in the presence of blood flow oriented parallel to the AE showed a decrease in diameter up to 21%, while in the case of a perpendicular location of the blood flow, the area was decreased by 9%.

According to Huang [15], the location of the vessel is essential to its cooling effects. A distance of 0–1 cm between the artery and electrode decreases the heating temperature depending on the vessel location. Moreover, a high flow rate of the blood and artery location (parallel and orthogonal) reduce the temperature by 5% at least. Another work of De Vita et al. [18] on microwave ablation also reported the impact of the presence of vessels near the antenna on the decrease in temperature. The parallel-located catheter has a heat-sink effect during the ablation. The study showed that a vessel placed 8.5 mm away from the tip caused heat dissipation. It demonstrated that the position of the vessel directly impacts the ablation process and treatment outcomes.

Figure 10 and Figure 11 demonstrated the heating impact contributed by the agarose solvent that can increase the temperature and area of ablation in a shorter period of time compared to the pristine tissue. Moreover, the agarose gel provided a similar heating trend for all trials with the minimal standard deviation values. This experimental analysis shows two important conclusions: (1) The use of agarose gel substantially increases the ablated surface, as the average values rise from 25–40 mm^2^ without agarose to 56–70 mm^2^ with agarose; (2) The agarose gel counters the heat-sink effect significantly, resulting in ablated areas that are even larger than in absence of blood vessels (55 mm^2^ average as reported in [12]). This can be explained by the fact that the medical tube has a resistance of a few tens of kΩ, but when coated with agarose gel, the impedance significantly lowers and allows heat to pass through; in this case, since we used a higher RF power, we could also obtain larger ablated surfaces. In addition, as reported in Figure 9, even the shapes of the thermal maps are well-preserved and do not appear to differ substantially from a round contour even in the presence of a blood vessel.

The proposed temperature-sensing network of Mg-silicate-doped optical fibers enabled the detection of changes in temperature with high precision in real time in the presence of the heat-sink effect. Moreover, the current study confirmed that optical fiber temperature sensors can detect changes in temperature with high precision in the presence of vessels in any positions. Hence, the explored outcome can contribute further to clinical studies where it is vital to be able to detect any temperature variations in order to prevent damage to healthy cells. Moreover, the heat-sink effect limits the complete ablation of a tumor; therefore, it is of great importance to monitor accurately the heating during RFA.

## 5. Conclusions

In conclusion, we presented a setup that mimics the dynamics of RF ablation applied to cancer care in the presence of a blood vessel, while simultaneously recording temperature maps over the tissue surface with a narrow spatial resolution (102 sensing points over 800 mm^2^), utilizing distributed optical fiber sensors. The blood vessel induces a heat-sink effect that reduces the ablated surface, experimentally. We recorded average ablation surfaces of 40 mm^2^ and 26 mm^2^ when the blood vessel is positioned in parallel and perpendicular to the applicator, respectively; the average cytotoxicity areas are 98 mm^2^ and 72 mm^2^, respectively.

We propose a solution to the heat-sink problem by injecting agarose gel in the tissue, which allows for more efficient heat transfer. In the presence of agarose, the ablated region extends to 56 mm^2^ (parallel) and 70 mm^2^ (perpendicular), while the cytotoxicity regions extend to 158 mm^2^ and 156 mm^2^, respectively.

As RF ablation targets solid tumors, such as hepatocellular carcinoma, that are often located in sites neighboring large blood vessels, the influence of heat-sink effects can significantly impact its performance and can largely go undetected when using single-point sensors such as thermocouples or few-point sensors such as FBGs. In this work, the proposed synergic approach incorporates agarose gel, biocompatible and injectable in tissue to facilitate impedance handling and heat transfer, and a fiber-optic sensing network that in real time renders the temperature distribution with high precision and a spatial resolution even narrower than magnetic resonance imaging. The approach has been experimentally validated, over a set of experiments in phantom in order to show its effectiveness both as a sensing unit, and as a method to extend the ablated surface up to 15% using the agarose gel injection.

The proposed method for temperature sensing and estimating the potential heat-sink effects has been designed in order to be applied in clinical settings. The fibers can at first be placed in the tissue using Chiba-type needles, that can then be removed in order to leave the fiber in place, while FBGs would need a placement relative to the blood vessel in order to render the heat-sink effect; the use of densely distributed sensors provides a much better thermal rendering without the need of a specific placement of each sensing unit. In addition, this method could extend the number of fibers, and therefore the sensing point, reducing density and increasing the dimensionality from 2D to 3D.

## Data Availability

Data presented in this work are not publicly available at this time, but can be obtained upon reasonable request from the authors. https://www.dropbox.com/sh/pf6tpyfezed2mfx/AACoLSqn1q0MHB_Ja1mY0FSla/Code/New%20maps%20ver.2?dl=0&subfolder_nav_tracking=1 (accessed on 5 December 2022); https://www.dropbox.com/sh/64c1cq0xb02mo8g/AAC1r1zuxFglBvJTnlUVFUp2a/Code/New%20maps%20ver.%202?dl=0&subfolder_nav_tracking=1 (accessed on 5 December 2022); https://www.dropbox.com/sh/5qa9rao7te4fbdj/AADD3U64yMdPfDnTTzeM9kcNa?dl=0 (accessed on 5 December 2022).

## Supplementary Materials

The following supporting information can be downloaded at https://www.dropbox.com/sh/pf6tpyfezed2mfx/AACoLSqn1q0MHB_Ja1mY0FSla/Code/New%20maps%20ver.2?dl=0&subfolder_nav_tracking=1; https://www.dropbox.com/sh/64c1cq0xb02mo8g/AAC1r1zuxFglBvJTnlUVFUp2a/Code/New%20maps%20ver.%202?dl=0&subfolder_nav_tracking=1; https://www.dropbox.com/sh/5qa9rao7te4fbdj/AADD3U64yMdPfDnTTzeM9kcNa?dl=0.
